# Editorial: Platelets in tumor biology: from molecular mechanisms to clinical applications

**DOI:** 10.3389/fcell.2024.1373463

**Published:** 2024-02-13

**Authors:** Mariana Aris, Anil K. Sood, Alexander Zaslavsky

**Affiliations:** ^1^ Centro de Investigaciones Oncológicas-Fundación Cáncer (CIO-FUCA), Buenos Aires, Argentina; ^2^ Department of Gynecologic Oncology and Reproductive Medicine, UT MD Anderson Cancer Center, Houston, TX, United States; ^3^ Department of Urology, Rogel Cancer Center, University of Michigan, Ann Arbor, MI, United States

**Keywords:** platelets, cancer, tumor biology, tumor immunology, angiogenesis, thrombosis, metastasis, biomarkers

Traditionally recognized for their pivotal role in blood clotting, in recent years, platelets have acquired increasing attention for their multifaceted contributions to inflammatory and immune responses (Morrell et al., 2019). While essential components of the circulatory system, platelet involvement extends far beyond clot formation, revealing complex mechanisms that maintain the body’s equilibrium. Platelets arise as anucleate cytoplasmic fragments derived from megakaryocytes, generating different mature mRNAs in response to external stimuli (Nassa et al., 2018). Platelet levels are increased in cancer patients, and they can coat circulating tumor cells (CTCs), releasing platelet-derived microparticles that promote metastasis (In’t Veld and Wurdinger, 2019). Platelets can be affected by tumor cells as well: the so-called tumor-educated platelets (TEPs) (Roweth and Battinelli, 2021). There is still much to learn about how this crosstalk could promote tumor growth and progression. For instance, the role of platelets as modulators of the antitumor immune response has been recently described (Chapman et al., 2012; Zaslavsky et al., 2020; Gockel et al., 2022). These emerging biomarkers in liquid biopsies might directly impact clinical practice.

This Research Topic contains four articles, namely, two original reports and two reviews, related to the role of platelets in cancer growth and progression. In their research article, Kelly et al. analyzed the role of PAI-1 in the progression of high-grade serous ovarian cancer, the most common subtype of epithelial ovarian cancer. The strategy involved PAI-1 gene-silencing of cell lines treated either with healthy donor platelets or platelet-conditioned media, followed by gene expression analysis, revealing that loss of PAI-1 disrupts pathways integral to metastatic processes. Furthermore, PAI-1 levels were reduced in plasma from patients following neoadjuvant chemotherapy. These data highlight PAI-1’s role as a potential biomarker of prognosis and response to therapy.

In the work submitted by Eslami-Set al. the crosstalk between platelets and tumor cells in colorectal cancer (CRC) was explored. In this case, the authors analyzed the effect of platelets and their conditioned media on cell lines obtained from circulating tumor cells (CTCs) of patients with CRC, followed by gene expression analysis. This study revealed that both conditioned media and cell-to-cell contact influenced the profile of platelets, promoting their activation and aggregation, and of CTCs, increasing the expression of genes involved in cancer invasiveness.

Li et al. reviewed the interactions of platelets with tumor and non-tumor cells during cancer development and progression. Platelets play an important role in triggering thrombosis and inflammation, linking hemostasis and immune responses. Platelets act on immune cells, contributing to either anti- and pro-tumor immune responses recruiting erythrocytes, monocytes, and macrophages. They can also promote maturation of dendritic cells and antigen presentation, stimulate neovascularization, contribute to formation of neutrophil traps (NETs), impair NK cell immune surveillance, and foster Th1, Th17, and Treg differentiation. Finally, Bekendam and Ravid reviewed the mechanisms of platelet activation in cancer-associated thrombosis, with a focus on myeloproliferative neoplasms (MPNs), characterized by clonal expansion of myeloid precursors and abnormal function of erythrocytes, leukocytes, and platelets, leading to thrombotic and hemorrhagic complications.

Platelets are increasingly recognized as key players in tumor biology and tumor immunology. The contributions included in this Research Topic have further advanced the current lines of investigation. More research in this field is needed to understand the full impact of platelets on cancer biology, leading to the discovery and implementation of biomarkers in clinical practice and the development of innovative therapies.
